# The complexity of architectural and anthropological dynamics in womb-tomb structures: An interdisciplinary investigation

**DOI:** 10.1371/journal.pone.0317058

**Published:** 2025-03-03

**Authors:** Nurit Stadler, Shlomit Flint

**Affiliations:** 1 Department of Sociology and Anthropology, the Hebrew University of Jerusalem, Israel; 2 Department of Geography, Bar Ilan University, Ramat Gan, Israel; Tel Aviv university, ISRAEL

## Abstract

Our research investigates a collection of distinctive case studies identified as ‘womb-tomb sacred structures,’ scattered throughout the region colloquially referred to as the Holy Land. This geographical area, nestled between the Mediterranean Sea and the East Bank of the Jordan River, holds profound historical and religious significance, intersecting with the biblical Land of Israel and the broader region of Palestine. It is revered by followers of Judaism, Christianity, and Islam. This study offers a pioneering exploration of the complex interplay between human corporeal rituals at sacred sites and architectural forms, illuminating not only how these rituals impact architectural design but also how the design influences the rituals themselves. We have catalogued an extensive array of womb-tomb structures across this landscape, dating from various periods including the First and Second Temples (700 BCE) and the Byzantine era. Despite their chronological diversity, these structures share architectural characteristics—typically, they are enclosed, cave-like spaces, often dimly lit and surmounted by domes. The architectural geomorphology of the womb-tomb designs prescribes specific bodily movements, with their distinctive forms necessitating, and sometimes enforcing, actions such as bending, crawling, and bowing within these enveloping spaces. Our exploration is supported by a novel methodological framework consisting of nine stages, which harmoniously blends architectural analysis (including Spatial Analysis, Morphological Analysis, 4D Approach) with anthropological perspectives and methodologies (including observations, interviews, photographic documentation, and short films). Our results elucidate that these architectural structures and morphologies serve not merely as sanctified sites but also unveil previously unrecognized connections among human anatomy, architectural configurations of the afterlife, and the connections between ritualistic conceptualization of soil, land, and territory as expressions of ownership and power.

## Introduction: persephone’s dilemma

The Greek myth of Persephone and Demeter mirrors the cycle of life, where the goddess Demeter descends into the underworld to save her daughter Persephone from the lord of death [1,2]. This tension between life and death, which is embedded in most world myths, is also apparent in worship in cave-like shrines, where followers enact rituals to seek fertility and good health. Historically humans have used subterranean spaces for survival, art, burials, and spiritual rites [3–5]. While research has indicated that the interplay between human actions and the environment in these rituals reflects the balance between life’s end and rebirth [6,7], there is still no comprehensive approach to exploring how both overt and covert infrastructures impact rituals in sacred spaces, and the specific movements of the human body within these sacred caves in particular.

This paper centers on an exploration of three pivotal concepts: ritual, form, and territory, within the context of anthropological and architectural discourse. Our aim is bifurcated. Firstly, to engage these concepts, as elucidated in scholarly literature and manifested in the practices and perceptions of pilgrims, in a critical dialogue and examination of their interplay. Secondly, to articulate these notions by delving into the intricate and emotive connections between land tenure, sacred architecture, and associated practices in the sanctified spaces of the Holy Land.

Building on studies that explore the interplays between constructed spaces, cultural landscape and human activities [8,9], we explore the influence of structural design on human movement within the architecture of sacred shrines. Specifically, we examined how sacred architecture evokes a profound ritualistic body experience. To do so, we developed a novel (9 stages) architectural-anthropological approach that examines the overt and covert infrastructures of womb-tomb sacred locales, by delving into their contemporary redesign, reconstruction, and the significance of these sites in shaping individuals’ sacred experiences. By merging anthropological tools, space analysis and rituals with architecture’s prowess in creation and innovation, we define a conceptual space that allows us to explore the fluid boundaries between visible and hidden infrastructures, cultural and physical, and the intricate interplay between humans and their built environment.

The concept of Architecture Without Architects [10] breaks down narrow views on the art of building by introducing the unfamiliar world of non-pedigreed vernacular, anonymous, spontaneous, indigenous or rural architecture. It includes communal architecture; i.e., architecture that is not produced but by the spontaneous and continuing activity of a whole people with a common heritage, acting within a community of experience. While we are reasonably well informed about the artistic objectives and technical proficiency of painters 30,000 years before our time and archeologists sometimes discover remnants of a town that date back to the third millennium B.C.., our grasp of the full picture of anonymous architecture is distorted by a shortage of documents, visual or otherwise. Rudofsky [10] pointed out that “the beauty of this architecture has often been dismissed as accidental, but today we recognize in it an art form that has resulted from human intelligence applied to uniquely human modes of life. “

There is also more to infrastructure than meets the eye. These hidden systems: e.g., mold, air flows, humidity, and narrow passages, are all equally important in understanding how people experience sacred places and landscapes [11–13]. We define hidden infrastructures as the systems that facilitate the circulation of goods, morphology, knowledge, meaning, people, materials, and power [14,15]. The conceptual notion of womb-tomb structures serves as the framework to investigate the cultural-geology and form of sacred vernacular architectures and better understand how their unique infrastructures guide bodily movements and acts of worship within these revered spaces.

The analysis of these sites centers on the area known as the Holy Land, which is roughly situated between the Mediterranean Sea and the East Bank of the Jordan River [16]. This area is traditionally associated with the biblical Land of Israel and the region of Palestine and is regarded as holy by Jews, Christians, and Muslims alike [17]. For this analysis we identified approximately 300 womb tombs in this area which, although built during different periods, including the First and Second Temples (700 BCE), as well as the Byzantine period, More than two-thirds of these sites are located in the Galilee, an area known for its rich historical and cultural significance across religious traditions. The sites are located west of the Sea of Galilee in an area known for its terra rossa and rendzina soils. Rendzina soils usually have a small soil volume thus making them impermeable to water. In addition, these soils often occur on slopes, in areas where there is considerable semi-natural vegetation. Terra rossa and rendzina soils were used as building materials in the past because they are soft and easy to shape. It is not uncommon for voids to remain in places where terra rossa and rendzina soils were mined to build nearby houses. Many of these spaces were later converted into burial sites. These, as well as the settlements in their proximity, reflect historical settlement patterns and are the sites of numerous archaeological findings. These pilgrimage sites tend to be located in accessible areas, and are concentrated in the Galilee region during the spring months when the weather is pleasant. Other sites are located near Haifa, Megiddo, and in Judea and Samaria, which are also characterized by terra rossa and radizmina rocks.

We term these burial sites womb tomb shrines [18]. They exhibit the same type of architecture that usually takes the form of enclosed, dimly-lit, cave-like structures, often covered with domes. Since these shrines often occupy cave-like settings, their front entrances are their lone gateway and the main (or only) light source. Because of their narrow, low entrances, visitors are forced to crouch and frequently rub against doors and entrances. The architectural geomorphology of womb-tomb designs mandates specific corporeal choreographies, with their unique forms necessitating and at times compelling actions such as bending, crawling, and bowing within the cavernous space. These spatial narratives and place-making elements embedded in the sacred topography facilitate a physical engagement that mirrors cultural and ritualistic practices. Our findings show that when pilgrims articulate their experiences within these sanctified landscapes, the embodied movements frequently converge with themes of fertility, sustenance, health, and a deep-seated sense of cultural landscape and land belonging, reflecting a human ecology intertwined with otherworldly connotations.

Lefebvre [19] argued that space is not neutral but is rather divided, organized and socially marked, thus creating a hierarchical system that directs individuals’ behavior. This rediscovery of the sensory experience in architecture [20–22] calls for the integration of qualitative and quantitative methodologies, and a combination of ethnographic tools with spatial, morphological, and 3D panoramic analyses of each site. This interdisciplinary approach can thus provide a new perspective on aesthetics that takes cultural and individual differences into account. Through the analysis of two sites, the tomb of Rabbi Bar Kappara and the tomb of Akashya, we demonstrate that body movements within the womb-tomb infrastructure allow visitors to engage in rituals that mirror the corporeal movement from birth to death and express a strong affiliation to the soil and the land.

## Literature review

### Inter- and intra-relations between architecture and anthropology

People do not only shape their surroundings such as their buildings and homes, cities, and landscapes, but are also shaped by them [23,24]. All of these interact with ways of inhabiting the world and intervene in where people belong and how space is lived. Calls have been made to develop new cultural-geography approaches combining architecture and anthropology [25–28] to better understand the connections between structure and human practice.

Vernacular architecture has been studied in many countries as a source of aesthetic inspiration for its functional, tectonic, and material properties, or as a foundation for general theories of architecture [10,29]. The conceptual rupture with modernism led to renewed interest in the social and cultural contexts in which architecture is integrated and coproduced, with a more recent emphasis on the importance of learning from native building techniques and local materials and developing them further [30,31]. Currently, this ethnographic turn can be observed in contemporary architectural and design research [32] which emphasizes that architecture is more than an object or a product, but rather a social and cultural process that involves both humans and non-human actors [33,34].

Early scholars such as Morgan [35] and Forde [36] put forward comparative theories that incorporated their knowledge of architecture and culture [37]. In anthropology, for example, architectural elements are discussed in Lévi-Strauss’ ‘Savage Mind’. He contrasted “bricolage” - the innovative reuse of materials - with the “engineering” typical of architectural standards to capture the relationship between culture and form Levi-Stauss [38]. Bourdiue [39] argued that architectural layouts reflect social structures that intertwine cultural practices with societal power dynamics and tastes. Latour [40] and others advocated for collaboration with informants, and encouraged ethnographic work to capture the complex societal relationships in their contextual structures and forms [41]. As a result, today’s anthropology acknowledges a broad spectrum of entities, from spirits to animals, caves to churches, as valid conversational partners.

The growing interest in actual and future users has led architects and planners to collaborate with anthropologists to develop conceptual strategies that take human behavior into account in built material environments [23,42–45], Despite the differences between the two disciplines [23], both architects and anthropologists use *concepts* and *models* to develop theories [46]. They complement each other when architecture asks what it ‘does’ to people, and anthropological methods are needed to answer such questions [47]. One of the most fruitful approaches to exploring intertwining human-material interactions, organisms, and objects was developed by Yaneva [48], who suggested six methodological steps: (1) adjust the pace of the inquiry and suspend judgment; (2) follow the paths and flows of non-humans and their connections; (3) remain on ground level since this is where little can be seen, but individuals can be seen by others; (4) learn from the visual epistemology of architects and mobilize design visuals as a form of generating knowledge; (5) revisit the descriptive techniques to better define the ontological granularity of architectural processes; (6) make a difference in the world of design. Although this approach provides strong inspiration and tools, it deals primarily with modern architecture and may not be sufficient to probe how infrastructure dictates sacred places, their mimetic rituals, human movement, and relationships with non-human beings.

## Sacred Architecture, Sacred Forms and Sacred Landscapes

The study of sacred architecture provides insights into the deep-rooted cultural, spiritual, and societal values of civilizations, as well as the intricate ways in which built environments can shape and reflect human beliefs, identities and spaces [49–53]. Sacred architecture does not just involve buildings as delimited designed objects, but also involves mold and microbes, walls and apertures, sounds and smells, legal and financial structures. Humans’ emotional responses and attachment to objects awakens the “sixth sense” of architecture and enhances conceptualizations of faith [54]. These can reflect the cosmological principles of a society, but they are also dynamic, complex landscapes [55,56]. For example, Ingold [57] suggested a methodology for examining medieval cathedrals. He shows how craftspeople and materials interact to generate form and suggested that researchers need to consider architecture as a mode of inquiry. In many cultures, structures are designed to convey spiritual connotations and religious identity [52], and thus utilize a specific language for the design and construction of places based on religious sentiments, rituals, and cultural aspirations [51,58].

Sacred spaces are some of the most powerful and impressive monolithic structures of humanity. As such, these places become spaces for pilgrims and visitors, where the sacred can be reflected, sensed, embodied, and translated into mimetic holy journeys. These occasions may include a set of rituals, worship, extravagant or impoverished materiality, and may have designated areas that can be visited by many or hidden from the public [59]. Koonce and Walker [60] suggested that sacred architecture should make “transparent the boundary between matter and mind, flesh and the spirit” [see also 61]. According to Kieckhefer [62], entering a religious building is akin to entering into a spiritual relationship, and constitutes a bonding with sacred elements. In *Theology in Stone*, Kieckhefer [62] defined three factors that affect the spiritual process and can be harnessed to analyze sacred space: longitudinal space which emphasizes the process and returns of sacramental acts, auditorium space which is suggestive of proclamation and response, and new forms of communal space designed to enhance intimacy and participation in worship on a minimal scale.

Buchli [27] examined the materiality of the built form through its material registers by examining space establishment and maintenance. He analyzed London’s Crystal Palace, ‘the ethnographic museum’ of the mid-19th century, and Bentham’s famous Panopticon to characterize the impact of institutional forms on anthropological thought, and argued that architectural form is the most important analytical category when probing the origins and notions of ideal forms in human society [27]. He suggested that architecture shapes, sustains (and unravels) social relations while giving meaning to people’s lives.

Consistent with the methodologies prevalent in architecture and cultural anthropology [63], we propose that womb-tomb structures epitomize a transcendental space, offering a unique lens to comprehend the intricate interplay between personal experiences of ritual, cosmologies and their spatial manifestation. These structures, encompassing chambers, caves, tombs, and narrow entrances/exits, not only embody the physicality of built environments but also resonate with the body movements in symbolic landscapes. They serve as a tangible interface where the sacred and profane, the metaphysical and the physical, converge, reflecting the cultural topography and the socio-spatial dynamics inherent in the ritualistic practices of a the visitors and local community [49,50,64,65].

## Sacred infrastructures and human anatomy

Womb-tomb shrines are universal and have a long history [66,67]. A womb-tomb infrastructure usually has a circular or elliptical shape within which is a central tomb or burial chamber [68]. Since the early Christian period, underground infrastructures have been used to represent themes such as the resurrection and eternal life [69,70] worldwide [71]. In Scotland and Ireland, Cochrane [72] found evidence that these structures were used as veneration sites as of the Neolithic. It has been suggested that in times of stress and infertility, the popularity of these places can be attributed to the way their architecture emulates the female womb [73]. The Aztecs worshipped in womb-tomb shrines, the most famous of which are the Teotihuacan’s two pyramids [74,75]. Old British Anglican traditions also included tomb shrines, such as the tomb of Edward the Confessor in today’s Westminster Abbey [76]. The Pantheon in Rome, which was originally built as a temple to the gods in ancient Rome, was later converted into a church in the form of a womb-tomb [77]. The circular dome and the central opening at the top, known as the “oculus,” symbolize the womb and the tomb, respectively. The circular design of the Holy Sepulcher in Jerusalem, which is believed to be built over the site of Jesus’ tomb, also follows this sacred archetype.

The connections between built settings and the human body have been discussed since the first century, when Vitruvius argued that the human body is the ideal model for all forms of architecture [78,79]. According to Vitruvius, buildings should reflect the perfect proportions of human bodies by emphasizing symmetry and balance [80–82]. In his 1525 treatise “Underweysung der Messung”, Dürer studied human anatomy in depth as a key to the symbolism of sacred architecture [78,79]. The fourth section of Dürer’s work discusses the relationship between the body and the Christian doctrine of resurrection to explain imitations of visual sacred models [78:68].

Insipired by these works Panofsky’s approach [78] also sought to understand how artists and architects used symbolism and iconography in the architecture of cemeteries and funerary monuments to express deeper intellectual and cultural meanings [82]. By incorporating death imagery and afterlife cosmology into tomb design such as skulls and skeletons, tombs reinforce religious beliefs, moral lessons, and life and afterlife ideals by illustrating life’s transience and inevitability. Gimbutas [83,84] considered that many ancient goddess figures in Old European cultures represented the “Great Goddess” who was associated with fertility, birth, death, and regeneration. James Mellaart examined the symbol of the son and lover of the Great Mother Goddess worshipped at the Çatalhöyük site in Turkey, which was inhabited between 7500-5700 BCE, and posited that oval-shaped houses decorated with images of the mother goddess symbolized the womb [85]. Krautheimer [86] suggested that these two symbols - the womb and the tomb - in early Christian architecture represented the belief in the resurrection of the dead as well as the hope for eternal life [87]. Coleman and Elsner [88] coined the concept of “body in motion,” which can account for current practices of devotion, body movement, and the use of the senses in sacred sites in underground infrastructures in womb-tomb settings [89] mimetic body-based rituals such as crawling, knee walking, bending, kissing, praying, touching, smelling and candle-lighting are thus all part of an experience that is replicated and standardized, which promote devotion to the womb-tomb saint along with the popularization of these shrines.

Based on these scholarly works, we presented a novel architecture-anthropology methodology that served to reveal unique structural and ritual relationships. The findings suggest that womb-tomb structures differ from other sacred spaces primarily on the basis of their architectural characteristics, which are intricately linked to the ritual practices that take place within them. Unlike many sacred sites in the Holy Land that are also situated in caves or enclosed structures, womb-tomb spaces are deliberately designed to evoke the sensation of entering a womb. We showed that this architectural intention is functional, not merely esthetic, in that it fosters a physical and symbolic journey that symbolizes a return to the origin and an encounter with the afterlife. The narrow entrances and dimly lit interiors of these structures are crafted to necessitate physical maneuvers such as bending, knee walking, crawling, or squeezing, which are emblematic of rebirth and transformation. These are mimetic rituals, in which the site shapes body movements in such a way as to reconnect with the territory while imitating the fetus coming out of the womb.

By contrast, other sacred sites such as synagogues, churches, mosques, and those located in open spaces like riversides, hillsides, trees, or open cemeteries, including cave-based sites such as Nabi Ukasha/Benjamin’s tomb in Jerusalem and Rachel’s tomb in Tiberias, although important for worship and veneration, do not typically require or facilitate such specific physical engagement. While these sites often center around grave worship, they lack the architectural elements designed to explicitly reflect the dual symbolism of womb and tomb. Our findings show that womb-tomb structures integrate both human-made and natural elements that enhance the ritual experience by utilizing the physical environment to embody and convey the profound themes of life, death, and renewal. The strategic placement of tombs within these caves or in compact rooms, combined with the encompassing architectural style, creates a distinctive experiential space that markedly contrasts with more open or traditionally structured sacred sites.

Thus, while many sites in the Holy Land are revered for their historical, religious, textual and mythological specificities, womb-tomb sites are uniquely characterized by their architectural and ritualistic features that are designed to immerse visitors in a transformative physical and spiritual journey. This interaction with space and symbolism defines the womb-tomb experience, and sets these sites apart from other types of sacred architecture across religious traditions.

This more fine-grained perspective shows how the interplay between architectural form, human kinetics, and spatial configuration transcends religious affiliation. The architectural design of womb-tomb structures precedes and dominates the contextual and political frameworks that later endorse specific religious uses. Our findings indicate that it is the structure that initially dictates the ritual activity; only later on do sociopolitical forces enable certain religious groups to adopt the site, endow it with a name, associate it with a venerated individual, and weave tailored mythologies that fit the spatial narrative. As shown here, the potency of architectural form is paramount, and exerts a more substantial influence than either phenomenology or religious cosmology. Despite differences in religious affiliations, the ritual practices facilitated by womb-tomb architectures exhibit remarkable uniformity, thus underscoring this structure’s dominant role in shaping ritual conduct across different faiths.

## Method and data analysis

Our methodology is a synthesis of interdisciplinary approaches, blending anthropological and architectural methods into a seamless, unified framework. This methodological fusion is articulated through nine distinct yet inherently interwoven phases. Our focus is on the intricate examination of body movements and ritualistic performances within sacred spaces. Central to this approach is the integration of spatial analysis, place-making concepts, and the study of cultural landscapes. This integration is not merely a collection of diverse tools; rather, it forms a cohesive and interconnected scheme essential for understanding the dynamic interaction between human activities and sacred environments. This study received ethical approval as part of the “Sacred Archetypes and the Architecture of Worship”BIUGEO_SFA_021223_1 (24.10.2023).

*Phase 1: Team Evaluation* The first phase involved forming a team of researchers, anthropologists and architects, who selected the womb-tomb sites in holy land landscapes. In the preparatory phase, a short questionnaire with questions about the site, its meanings, symbols, and material objects were devised. The team then chose specific informants, men and women, from a range of religious denominations, ages, backgrounds, places of birth, and ethnicities. To better understand the users’ movement patterns, movement radius, and space available for visual and physical contact, the team defined several key points for each site based on their location and role within the site (entrance, main gathering point, etc.) and examined them using anthropological tools along with objective measurements of the space, to define patterns of movement within the womb-tombs for both males and females. In general, views run along the cave’s primary axes and secondary axes, and aim to cover historic frontages, and forms of ritual activity. However, the views and quality of the space observed from different locations can differ as a function of the morphological conditions of the surroundings.

*Phase 2: Criteria for the selection of places* To date, we have identified and collected materials in more than 300 womb-tomb shrines in the Holy Land to define their basic womb-tomb components. The narratives associated with these sites are inspired by the three canonical books, and all met the following criteria: a. They commemorate a holy biblical, canonical or mythological figure; b. They are open to the general public; and c. They have a womb tomb-form with a small entrance, tomb or tombs in the center, a round roof, a small dome and a grotto. Aside from these shared parameters, the sites were far from homogenous. To explore these differences, we contextualized these sites and situated them in terms of their eras of construction, specific socio-historical and political circumstances, and current mythologies. We drew up the following preliminary typology: a. Traditional well-established popular shrines; b. Local village shrines; c. Grotto shrines; d. Renovated shrines; and e. Abandoned shrines. This typology helped determine the central locations and shrines for the development of our theory and methods.

*Phase 3: Data collection and digitalization* During this phase we collected documentation on each shrine and analyzed it in time and space to better understand the processes that characterize womb-tombs along their main axes, water sources and settlements. This data also included information about the identities of their users and the “cycles” of each womb-tomb across religions and cultures. The output provided us with a detailed anthropological-architecture picture of the experiences, rituals and development of the built environment of the sites.

*Phase 4: Spatial Analysis and Map Production of the Sites:* This phase consisted of generating spatial distribution maps for the sites/typologies (R and ArcGIS) and aggregating background data related to previous and current access routes, the pathway network and the settlements. The database was geo-referenced to the parcels’ GIS layer (https://www.openstreetmap.org/searchquery=Bar%20Kappara%20grave#map=13/32.8522/35.4576&layers=P, open source, with residential, infrastructure, topographic, and land ownership data, updated in April 2022). The layers were compared to aerial photographs which are available for almost every decade since the 1950s.

*Phase 5: Participant Observations and Interviews:* This phase consisted of conducting observations and short interviews on each site, which allowed us to map the area in terms of type of movement/users and the creation of meaning. To complement the observations and interviews we also took photos and made short films. These observations were used to define the key locations for the morphological analysis of locations where most of the interactions take place. We observed and documented the sensory experiences at the shrines in detail by implementing a kinesthetic analysis [90–92]. This approach involved detailing the visitors’ body movements and rituals as they entered and prayed within these specific womb-tomb structures [91]. Our notes and descriptions covered the sites’ rituals, bodily performances, conduct, and elements of nature, ranging from soil and trees to flowers and stones. The interviews centered mostly on 1. Background questions about age, place of birth and residence, religious denomination, ethnicity and occupation, 2. The reasons for their visits to the specific shrine, 3. The visitors’ bond with the local saint. The goal was to uncover the significance of the saint in their lives, 4. Knowledge of the pertinence of specific canonical texts, narratives, and mythologies related to each site and 5. A short description of their experience at the shrine.

*Phase 6: Morphological analysis:* To account for the connectivity and the integration of the womb-tombs’ architectural and behavioral spaces and their users, we conducted a spatial analysis using the isovist analytical technique [93]. A single isovist is the volume of space visible from a given point in space, together with a specification of the location of that point [94,95]. An isovist can be considered the volume of space illuminated by a point source of light. Every point in physical space has an isovist associated with it. The resulting isovists determine visibility and accessibility to or from a particular point within emerging morphological arrangements (see also Fig 3a).

*Phase 7: Developing a 4D Approach* Based on the previous stages, which provided us with information about the ways in which the socio-spatial organization affects religious experience, we combined the methods to better understand how body movements shape the womb-tomb space over time, and how this experience is intensified. The 4D space-time analysis makes it possible to shed light on the ways the human body adapts to space, unlike the interviews, which were designed to distinguish between the different movements of female and male bodies as the source of the active dynamic experience. The tight integration of anthropological-architecture methods for analyzing spatial layouts and human activity patterns in enclosed sites helped decipher the impact of activities involving the user indirectly when reaching the goal room by specifying where people are, how they move, how they adapt, how they change and how they talk about it.

*Phase 8: 3D panoramic analysis* We constructed a set of two-dimensional plans in the horizontal section and at least four sections of each site. 3D visualization of the cave bought by Mabat 2023 (https://mabat3d.com/); Compilation of a section bought by Mabat depicts the users’ movement in space, and isovist analysis showing the adaptation and partial transformation of the spatial structure over time. Utilizing the Navisworks Roamer 3D Viewer, we reviewed and navigated around it in real time (4th dimension). Photorealistic images and animations, material estimates, measurements, and womb-tomb components were obtained after adding data and lighting to the model.

*Phase 9 Testing and enhancing the 4D anthropological-architecture approach* To create one complete set of tools, the Unity and Navisworks 3D Viewer was employed as 3D assessment software. It can track “real” processes and not only represent multiple time-states; e.g., in a 3D axonometric set(s) of floor plans. In the future this will make it possible to apply the approach to all 300 + womb-tombs identified in the Holy Land in a comparative manner. The tools can then be applied to identify the relationship between human-space movement and the creation of the sacred place.

## Results

### Shrine infrastructures and the human anatomy

Utilizing our nine-stage methodology, we have elected to focus our research on two specific cave structures: the tomb cave of Rabbi Kappara and his sons, and the tomb of Rabbi Akashia. These sites have been selected due to their exemplary representation of sacred womb-tomb locations, offering us a unique opportunity to apply our methodological approach. We chose these sites not for their Jewish symbols or associated mythologies but for their structural features. We posits that Jewish, Muslim, or Christian meanings, symbols, images, narratives, texts, and mythologies were later imposed and adapted to the site, and were transformed by contextual, political, and agency-driven factors. The structural aspects of womb-tomb architectures exert a stronger influence than the religious affiliations they later accommodate, since they are shaped by the historical and cultural contexts in which these structures are situated. The womb-tomb architecture facilitates a unique form of engagement which, while influenced by the specific religious traditions of the communities that frequent these sites, supports a range of ritual practices that transcend a single religious cosmology. These practices are universally influenced by themes of birth, death, and rebirth that resonate across different faiths.

This article centers on two sites that are currently defined as Jewish, to illustrate how these universal themes are manifested within a distinctly Jewish cultural framework. These sites exemplify the processes we aim to highlight where the dominance of Jewish actors, the state, State agents, political forces, and local agents collaboratively contribute to the Judaization of these sites. Thus, if these sites were under a Muslim (or any other) state, they would likely be dedicated to Muslim (or other) saints and frequented by Muslim (or other) pilgrims.

These two caves are located 16.8 km from each other, and can be reached by car in half an hour. In ancient times it took three hours by foot or about an hour and a half on a donkey along the trail that winds through a natural hillside environment. Although the historical narrative remains unknown, legends linked to the two caves can be associated with the broader Judaization of this area.

## The cave of Rabbi Bar Kappara

**Fig 1:**
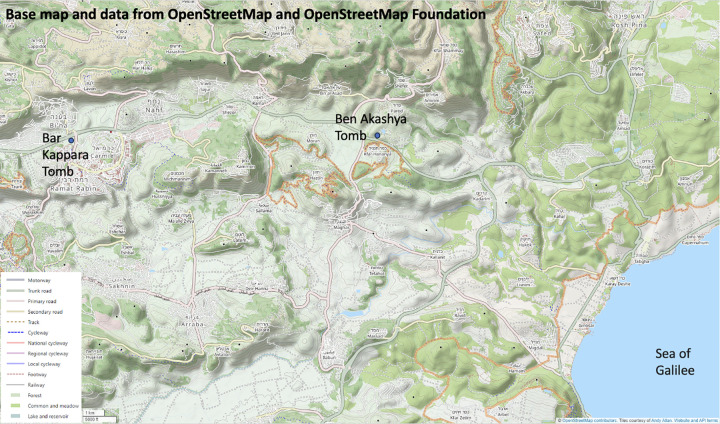
Location of Rabbi Bar Kappara’s and Ben Akashia’s womb-tombs.

**Fig 2:**
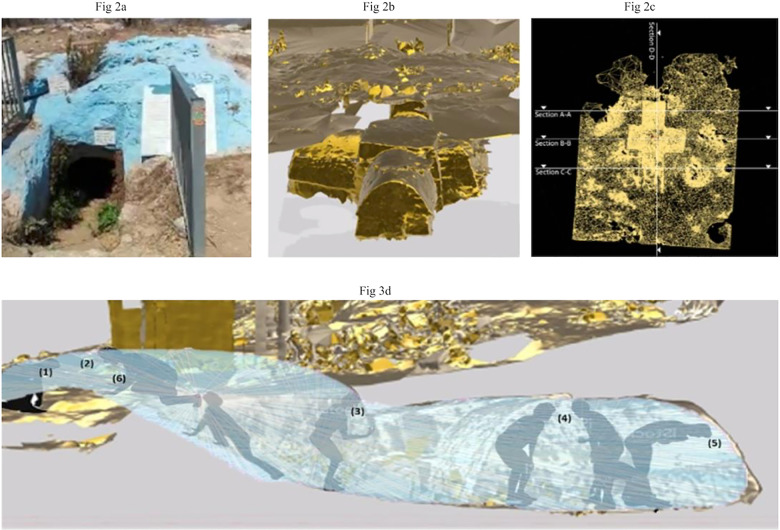
An analysis of the womb-tomb of Bar Kappara.

Located in the Galilee (Fig 1), the burial cave dedicated to Rabbi Bar Kappara (Fig 2) was rediscovered in 2001, and the site is associated with Mamluk and Ottoman periods. It is appropriated by a local ultra-Orthodox community who identified and marked it as the grave of the rabbi and his sons [96]. When we asked visitors about the Bar Kappara family (aka Bar Capra and Rabbi Elazar HaKfar), the few who knew the legend told the story of a Jewish sage who lived during the transitional period between the Tannaim and the Amoraim, around the 3rd century CE. He was known for his wit, his scholarship, and sometimes for his strained relationship with certain other rabbis, notably Rabbi Judah HaNasi. Visitors who were more knowledgeable told us that Bar Kappara was unique because he combined scholarly pursuits with humor. According to tradition, he was not just a legal scholar but also a poet and fabulist. His fables often incorporated moral or ethical teachings and were sometimes used to subtly criticize certain aspects of society or individuals [96].

When entering the cave, the natural underground formation depicted in Fig 2a-c provides visitors with an opportunity to interact with various natural elements. The entrance opens into a passage-shaped chamber, which like many other womb-tomb structures, mirrors the female reproductive system (Fig 2d). Perpendicular to this primary (southwest to northeast) axis—frequented by Jewish visitors—a secondary human-made grotto axis forms a cross-shaped grotto (Fig 2b-c). One assumption which needs to be verified is that Christian devotees once dug this passage and worshipped in this space. Today, this secondary axis is used for storage, thus orienting visitors to the main axis, which remains the only working passage.

**Fig 3:**
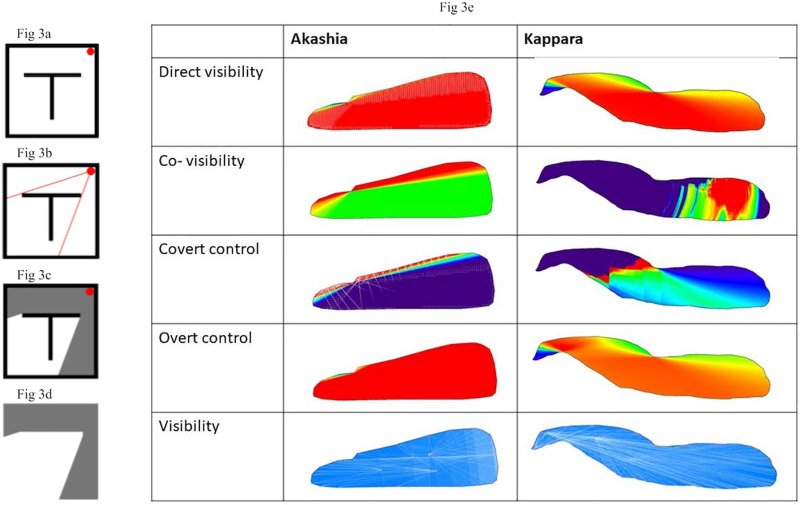
(a) Left: Isovist construction: (1) illustrative layout with a generating point for the isovist; (2) sight boundaries; (3) area visible within the sight boundaries; and the (4) resulted isovist; (b) Right: Visibility and control levels on both sites.

Following the Isovist construction (Fig 3a), and upon entering the narrow confines of Rabbi Bar Kappara’s tomb, visitors find themselves enveloped by the cave’s womb-like contours, with its water, vegetation, and distinctive geological features, including rock formations, stalactites, and stalagmites. This point has low directed visibility, such that it is difficult to see the entrance from other areas (Fig 3b2). People entering the tomb explain that this immersion into both the natural and human-made components dating back to antiquity enables a profound, symbolic and concrete union of the visitors’ bodies and their surrounding environment. The cave’s geological, natural, and constructed attributes can be seen as a tangible reflection of cosmology, sensual experiences, and ritualistic kinetic movement. These relationships concretize the integration of the body with these composite materials, the natural with human physicality, and the profane with the sacred. The outside world fades away, and is replaced by the cool, damp air and the muted echoes of dripping water. The walls, often slick and cold to the touch, are the bearers of the stories of millennia, transversed with mineral flecks and designs and sculpted by the ceaseless work of water. Shadows dance subtly with flickering light sources, adding a touch of mysticism to an environment that feels both ancient and untouched. The visitors, just like a fetus in the womb, must conform to the cave’s structure and features. Visitors need to bend, stretch, and contort their bodies to enter its constricted entrance, narrow small chamber and grotto. As visitors move along the main axis, the directed visibility increases and turns into co-visibility (Fig 3b4); i.e., the extent to which an individual can see and is seen, thus augmenting the area visible within one visual step from a location [97]. This contrasts with the low co-visibility at the entrance/exit where visitors see but are seen less than in the cave’s interior.

Similar to other natural caves that evoke feelings of protection, shelter, and solitude, the inherent darkness and echoing acoustics instill a deep sense of otherworldliness and corporeal intimacy with nature. Visitors say that rituals and prayers to the sages inside the cave underscore Earth-based spirituality, a strong connection with nature, and a feeling of transcendence in this connectivity. Visitors experience nature at its most ornate, composed of stalactites, columns, flowstones, draperies, cave curtains, rimstones and helictites. These natural materials and formations not only offer a glimpse into geological history and processes, but also create a mesmerizing underground landscape.

We compared covert control (Fig 3b6), which expresses the potential for any location to visually overlook others while avoiding scrutiny [93] to overt control (Fig 3b8), which is defined as the visual ‘linking’ dominance of any location [98] to examine ritual performance when individuals know that they are being watched in a public place as compared to situations that enable privacy and observation. The distribution and number of key points in the depths of the cave are lower than in the front, indicating their different functions (Fig 3b10). Individuals in the front have more covert control because in this location they can see regions of space that are better connected, and vice versa. In contrast, when a location has no visual access to regions that ‘see’ relatively less space than it does, overt control decreases, allowing individuals in the inner part to gather internally and detachedly. Lea (pseudonym, age 37, a mother of 4) described reading holy books by candlelight in this natural cave surrounded by burial mounds as a powerful spiritual-body based experience:

I am frequently drawn to these sacred caves and tombs that honor a sage who is important to me. I seek their quietude for reflection and meditation. I’m enamored with the enveloping darkness, the feeling of seclusion amidst nature and vegetation. It evokes the era of the Tanaim for me. The coziness and intimacy of the space make me feel as though my prayers are being channeled directly to their intended destination. I experience an overwhelming sense of belonging here as though the place embraced and absorbed me.

Within the cave, one of the most conducive posture for visitors to engage in prayer and worship is kneeling. The presence of scattered candles and prayer books in the cave and scattered around symbolizes ancient Jewish traditions and local mythologies of saints. Lit subtly by candles or subdued lighting, these spaces foster an intimate ambience that focuses the visitor’s attention on the central ritualistic elements and their own sensory experience. The natural structure (non -human) and architecture (human) emphasizes bodily engagement, the shades of light and darkness, humidity, the smell of soil and running water. The overarching ambience of the womb-tomb ritual space profoundly influence the individual’s emotional state, by eliciting feelings of awe, reverence, and at times trepidation.

### The Cave of Hananiah Ben Akashia

**Fig 4:**
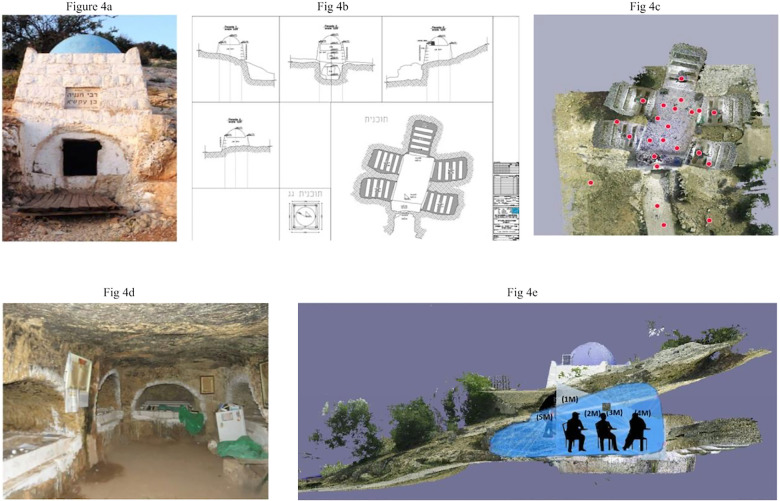
An analysis of the womb-tomb of Ben Akashia.

The second compound is associated with Hananiah Ben Akashia and is part of a complex housing the graves of other Tanaim and Tzadikim, including Rabbi Halfata and Rabbi Eliezer ben Ya’akov (Fig 4). The grave of Rabbi Hananiah ben Akashia is located near the village of Hananiah, south of highway 85 near the Halfata intersection. It is marked by a cave carved into the mountainside, enclosed by a stone framework (Fig 4a). Above this cave is a small stone edifice crowned by a blue dome, a conspicuous element contrasting with its environment [99]. Rabbi Moshe Basula provided a description of this tomb in his notes in 1521: “... above it is a cave, where Rabbi Hananiah ben Akashiya and his disciples are interred. I ventured inside to offer my prayers and observed four domes. Under each dome lie five graves, arranged facing each other [100]. A stone door can be shut at the cave’s entrance...”. Despite the historical references and the symmetric placement of the graves, the configuration of the graves, resembling a double cross, suggests possible Christian influence or use of the cave.

This natural cave has been altered through the years by human activity. A significant effort has been made to ensure that the visitors are able to resonate with the geometry, form and architectural design of the cave mixing the physical with the spiritual. During rituals, our findings suggest that the structure, not the script, establishes the type of body motion, mood and emotional resonance (Fig 4b-c). Despite the signs of human excavation, the folding plastic chairs and the white freshly painted graves, the darkness, the humidity, and the scent of the moss emphasize the feeling of being surrounded by a spiritual environment that includes the ground, water, minerals, cave stalactites, columns, and energy sources, as well as manufactured and consumed products such as food, clothing, technology, and infrastructure (Fig 4d).

The tomb complex is accessed via a narrow, human-made entrance that requires people to duck, as shown in Fig 4e. Upon entering, visitors are immediately enveloped in a cool, silent darker embrace, in stark contrast to the world outside. The dimly lit interior, with its ancient walls of sedimentary rock, prompts a sense of reverence, as many have described. Stalactites hang delicately from the ceiling, while the ground below reveal hints of metamorphic bedrock. As Moshe, a 25- year old Haredi from Tiberias indicated:

Every step I make echoes softly, reminding me the weight of history and the stories buried within layers of stratification“. The air has a musty scent, reminiscent of age-old stone and mineral deposits. As one ventures deeper into the cave-tomb, the geological formations become more pronounced, with a mixture of old and new reinforcing a palpable connection to the past and leaving an indelible impression of awe and reflection.

In Ben Akashia’s tomb, co-visibility (Fig 3b3) is created by the seamless integration of human-made physical forms (e.g., the door, the stone, the grave) and high direct visibility (Fig 3b1), which intentionally blurs the lines between human anatomy and the physical architecture. Upon entering the room, visitors are plunged into a dark environment (Fig 3b9) that requires them to adapt. Although human involvement in the excavation is evident, the overt and covert control (Fig 3b5,7) in the cave makes it a public domain.

Rachel (pseudonym, age 43, a mother of five) elucidated how engaging in personal prayer within a public space provides a wellspring of opportunity for personal renewal and a reconnection to the experience of rebirth:

To truly connect here, one must enter this space and let go. It feels like in a womb, drawing you back into childlike innocence, urging you to commune with your ancestors. Touching the stones, the tomb, the sacred texts, I feel reborn in a spiritual sense... This sensation of rebirth within the confines of this sacred place underscores the transformative power of prayer, symbolizing a return to a state of purity and spiritual awakening akin to the innocence of infancy.

The dozen or so visitors we interviewed entering the cave all indicated, as Rachel did that their time in the cave felt like a return to the womb. This experience provides them with insights into their own birth, as well as memories and sensations from their forgotten early lives. Through this journey, the body emerges as the primary sensory conduit to space and experience. The bond is further enriched by mimetic rituals deeply rooted in specific movements and intricately related to local myths and saints.

Analysis of the tomb cave of the sons of Kappara and the tomb of Rabbi Akashia reveals the intricate relationship between humans and their constructed environments as a persistent interplay. This intertwining of humans with their non-human surroundings underscores the notion of humans as influential geological forces [101]. The perspective offered by architectural-anthropology extends beyond traditional boundaries, encompassing elements like walls, views, sounds, and smells, as well as legal and financial structures that affect the way people inhabit the world. While some visitors may be somewhat familiar with the Tanaim narrative, the ceremony within the cave resonates deeply, prompting active participation, and becomes an experience they share with family and friends. To fully grasp the interplay between structure, geography, and ritualistic practices, and to understand the consumption of space, it is essential to meld anthropology’s profound insights with human behaviors and social relations with the prowess of architecture in shaping people’s living environments.

## Discussion

This paper introduces a mixed methodology for understanding the relationship between built environments and human body kinetic movements, focusing on womb-tomb shrines. We integrate anthropology and architecture to examine how these sacred spaces shape bodily movements and associated rituals. Our analysis begins with the interplay between structural design and physical movement, using concepts like spatial syntax and human-environment interaction. We then explore the symbolism, sense of place, and practices in these shrines, considering the cultural landscape, place identity, and phenomenological experience of space.

Our approach examines the blend of natural and human-made elements in these environments, emphasizing the traditions and intentions behind their designs. We employ analytical tools with an ethnographic lens to understand the interaction between space and human activity, focusing on the sacred archetype of womb-tomb structures within a geological context. This contributes to architectural anthropology by analyzing the interconnection of humans, the non-human, and the built environment. We define womb-tomb shrines and their infrastructures, combining structural analysis and a focus on embodiment and social relations to the land. This method proposes a theory of architectural anthropology, highlighting the interplay between physical structure and body movement in shaping religious experiences.

The contemporary architectural anthropology approach we adopt focuses on people, actions, and contexts, emphasizing the performative nature of knowledge practices. It is based on the Vitruvian Man concept, connecting architecture, anatomy, body movement, symbolism, and form. To understand movement patterns in case study sites, we used anthropological tools and the isovist technique. Our findings show that structures and the built environment shape religious experiences more significantly than legends or mythologies. Womb tomb shrines influence visitors’ movements, resonating with themes of fertility and life cycles. In the project’s second phase, we incorporated human narratives to understand their connection to the structure-body relationship. This Marxist materialist perspective reveals a profound bond between visitors and their physical and societal contexts. The physical attributes of the womb tomb often overshadow other influences like myths or traditions. Our interdisciplinary approach merges architectural and anthropological methodologies, offering a dynamic perspective on sacred spaces and rituals. This methodology enhances understanding of the relationship between constructed environments and rituals, emphasizing the role of architectural designs and natural elements in shaping ritualistic body movements.

With regard to gender, much more research needs to be done. We found that in the womb tomb structures we investigated, men and women engage similarly in practices and rituals as dictated by the form of the cave and other physical features. In other words, they bend, crawl, pray, etc. guided by the structure. However, there were subtle differences in certain phenomenological respects. Women often described their experiences in womb-tomb caves in terms of renewal and personal reflection, which align with the symbolic representation of birth and rebirth. Men, on the other hand, frequently focused on the mythological and textual significance of the site, thus emphasizing a connection with their ancestral and religious heritage. These differences underscore a gendered dimension to the engagement with sacred spaces, where architectural elements resonate differently with individuals based on gender, and influence how they experience and interpret the spiritual journey within these caves.

In sum, this study is among the first detailed explorations of womb-tomb sites, revealing deep connections with themes of fertility, life, and rebirth. By focusing on human narratives and mythologies, we show how sacred spaces encapsulate existential themes. Rituals at these sites symbolize life’s cyclical phases, highlighting the influence of sacred spaces on the continuum of existence.

## Additional information

**Places we have identify as Womb Tomb and are currently under investigation:**

a. Rabbi Elazar haQappara and his son Bar Qappara
b. Rabbi Yehoshua d’Sakhnin
c. Rabbi Hananya ben ‘aqashya
d. Kever Hillel Hazaken
e. Rabbi Yosi Ben Kisma
f. Ramban and the Tosofot (Tosafists)
g. Rab Hamnona Saba
h. Rabbi Zeharya Ben Hakatzav
i. Makom HaIdra Raba
j. The Tomb of Hana and her sons
k. Tomb of hosha Ben Beeri
l. Rabbi Eliezer Ben Yaakov
m. Rabbi Nechemia Haamsoni
n. Hanina ben Dosa
o. Rabbi Yaakov Birb
